# Gait, cognition and falls over 5 years, and motoric cognitive risk in New Zealand octogenarians: Te Puāwaitanga o Nga Tapuwae Kia Ora Tonu, LiLACS NZ

**DOI:** 10.1186/s12877-020-1420-8

**Published:** 2020-02-05

**Authors:** Sue Lord, Simon Moyes, Ruth Teh, Waiora Port, Marama Muru-Lanning, Catherine J Bacon, Tim Wilkinson, Ngaire Kerse

**Affiliations:** 1 Health & Rehabilitation Research Institute, School of Clinical Sciences, Auckland University of Technology, Auckland, 1142 New Zealand; 2 Department of General Practice and Primary Health Care, Faculty of Medical and Health Sciences, University of Auckland, Tamaki Campus, Auckland, New Zealand; 3 James Henare Māori Research Centre, University of Auckland, Auckland, New Zealand; 4 University of Otago, Christchurch, New Zealand

## Abstract

**Background:**

Understanding falls risk in advanced age is critical with people over 80 a rapidly growing demographic. Slow gait and cognitive complaint are established risk factors and together comprise the Motoric Cognitive Risk Syndrome (MCR). This study examined trajectories of gait and cognition and their association with falls over 5 years, and documented MCR in Māori and non-Māori of advanced age living in New Zealand.

**Method:**

Falls frequency was ascertained retrospectively at annual assessments. 3 m gait speed was measured and cognition was assessed using the Modified Mini-Mental Status Examination (3MS). Frequency of MCR was reported. Gait and cognition trajectories were modelled and clusters identified from Latent Class Analysis. Generalised linear models examined association between changes in gait, cognition, MCR and falls.

**Results:**

At baseline, 138 of 408 Māori (34%) and 205 of 512 non-Māori (40%) had fallen. Mean (SD) gait speed (m/s) for Māori was 0.66 (0.29) and 0.82 (0.26) for non-Māori. Respective 3MS scores were 86.2 (15.6) and 91.6 (10.4). Ten (4.3%) Maori participants met MCR criteria, compared with 7 (1.9%) non-Māori participants. Māori men were more likely to fall (OR 1.56; 95% CI 1.0–2.43 (*P* = 0.04) whilst for non-Māori slow gait increased falls risk (OR 0.40; 95% CI 0.24–0.68(*P* < 0.001). Non-Māori with MCR were more than twice as likely to fall than those without MCR (OR 2.45; 95% CI 1.06–5.68 (*P* = 0.03).

**Conclusions:**

Māori and non-Māori of advanced age show a mostly stable pattern of gait and cognition over time. Risk factors for falls differ for Māori, and do not include gait and cognition.

## Introduction

Falls in older people are burdensome and their impact is far-reaching. The effect of even a sporadic fall can be significant, especially for adults of advanced age. Falls are the third most common cause of accidental injury-related death globally, and the dominant cause of injury-related disability-adjusted life years (DALY) in older people [1]. Compounding this issue is the rapid demographic change with the number of adults aged 80 years and over projected to triple by 2050, rising globally from 137 million in 2017 to 425 million [2]. This age group is most vulnerable to the effect of falls*.* In New Zealand alone people aged 85 years and over experience 8 times more hosptal attendances than those aged 65–74 years, with 8.5% admitted as a result of a fall [3].

Falls are heterogenous and complex. They occur ultimately because of a failure to respond rapidly and effectively to perturbation during walking or when transitioning from one position to another Discerning the cause of failure is difficult because it is multifactorial. Numerous risk factors for falls have been identified such as previous falls, low self-efficacy, polypharmacy, and urinary incontinence [4]. Gait and balance (postural control) disorders are consistently implicated as prominent risk factors [5, 6] and restoration of both through exercise is integrated into falls prevention programmes [6]*.* Cognitive impairment is also an independent risk factor for falls, reflecting a key role for cognition which modifies both gait and postural control [7]. Cognitive retraining is therefore often integrated into falls prevention programmes, as is use of cholinesterase inhibitors to improve cognitive function [8]*.* Understanding the impact of gait and cognition on falls is important to improve early recognition of the potential for falls and inform effective prevention, which is challenging to implement and subject to failure [6].

The interaction between gait and cognition is also characterised as the Motoric Cognitive Risk (MCR) syndrome which is a pre-dementia state comprising slow gait and subjective cognitive complaint [9]*.* MCR syndrome is prognostic of both dementia [10] and falls*.* Callisaya and colleagues recently reported an increased risk of falling in adults classified as having MCR syndrome in over 6000 older adults from 5 longitudinal cohorts, likely due to the multiplicative effect of gait and cognitive complaints [11].

The trajectory of gait speed and cognition, and their relative contribution to falls in adults over 80 years has not been studied previously and is the focus of this research. In this prospective study we hypothesised that: 1) gait and cognition would decline over 5 years; 2) both would be independently associated with falls risk; and 3) MCR prevalence rates would be comparable to international reports.

## Method

### Recruitment and sampling

The study was embedded in a 5-year longitudinal research which commenced in New Zealand in 2010: Te Puāwaitanga o Ngā Tapuwae Kia Ora Tonu/Life and Living in Advanced Age: A Cohort Study in New Zealand (LiLACS NZ). The overarching study aim was to determine predictors of successful ageing and to understand trajectories of health and well-being for Māori and non-Māori of advanced age, in line with the global drive to study ageing. Study methodology has been described in full previously [12, 13]. Briefly, this study was part of a population based longitudinal study involving Māori (indigenous New Zealanders) and non-Māori octogenarians. Potential participants were identified through the New Zealand electoral roll, Primary Health Care data bases, Hauora (Māori Health Services), and community outreach. Exhaustive sampling was attempted where an attempt to contact and enroll every eligible person was made. LiLACS NZ recruited 937 participants from the Bay of Plenty and Rotorua regions of New Zealand in 2010–421 Māori born between 1920 and 1930 (aged 80–90 years, 56% of those eligible) and 516 non- Māori born in 1925 (aged 85 years, 59% of those eligible) [14]. All participants gave informed consent and The Northern X Regional Ethics Committee of New Zealand granted ethical approval for the longitudinal study in December 2009 (NTX/09/09/088).

### Measures

Participants were assessed at baseline and annually to 5 years follow up by trained interviwers using standardised techniques. A comprehensive assessment battery was administered via a home-based interview, followed by a physical and general health assessment [13]. *Falls frequency* was measured retrospectively via self-report. Participants were asked: How many times have you fallen in the past 12 months? A fall was defined as ‘unintentionally coming to rest on the ground or other lower surface without being exposed to overwhelming external force or a major internal event’ [15] *Gait speed* was measured over 3 m as part of the Short Performance Physical Battery. Participants were asked to walk at a comfortable pace for 2 trials, and the fastest speed was recorded [16]. *Cognition* was measured using the Modified Mini Mental Status Examination (3MS) which includes 4 extra items and is more nuanced in terms of memory items compared with the original MMSE [17, 18]. Depression was measured using the 15 item Geriatric Depression Scale (GDS) [19]*.* Participants met the criteria for *Motoric-Cognitive Risk (MCR) Syndrome* if they presented with a gait speed slower than 1 SD below the cohort mean as well as subjective cognitive impairment, indicated by a positive response to a question from the Geriatric Depression Scale *‘Do you feel you have more problems with your memory than most?* Both criteria were used by Verghese and colleagues in the original MCR defintion [8]. Frequency and amount of physical activity were measured using the 12-item Physical Activity Scale for the Elderly (PASE). Scores range from 0 (no activity) to a possible 400 [20]. Almost all answered a core set of questions including falls. About 2/3 Māori and 3/4 non-Māori answered a full questionnaire including gait speed and cognition.

#### Data analysis

Māori and non-Māori cohorts were analysed separately. We did not compare outcomes by gender given the limited data set for fallers especially in later years, but did include gender in final models (see below). Cognition (3MS) was converted to logit of the score due to its distribution which was extremely left skewed. Baseline demographic, clinical data and presence of MCR syndrome were reported. Differences between Māori and non-Māori were examined using Students *t-*test and the χ2 test as appropriate. Trajectories of cognition and gait were modelled over 5 years and Latent Class Analysis was used to classify participants into exclusive groups or clusters based on longitudinal gait and cognition scores. Frequency of participants in each gait and cognition cluster was described. Two models examined the association between gait, cognition and falls over the 5 year study period using Generalised Estimating Equation (GEE). For both analyses, falls were entered as the dependent variable and considered as *any prospective fall* over the duration of the study rather than restricted to a single year. For the first model (the ‘gait & cognition’ model) age, gait and cognition were entered as time-varying variables and gender as a fixed variable. The second model (the ‘MCR’ model) tested main effects and interaction effects for MCR, defined as the combination of subjective cognitive complaint and slow gait according to the thresholds described above. Independent variables included gender (male as the reference), age, gait speed, and cognition (logit of 3MS scores) and 2 gait*cognition groups: subjective cognitive complaint and normal gait; and no subjective cognitive complaint and slow gait. This enabled us to discern any differential effects of the gait*cognition interaction on falls. GEE confers advantages over other methods for analysing longitudinal data in that it does not penalise for missing data and it accommodates data that is not normally distributed. A criterion for using GEE is that a within-subject correlation structure must be selected prior to analysis [21]. We used an auto-regressive correlation structure, which denotes that observations on the same individual 1 year apart are higher than those on the same individual 2 years apart. Parameter estimates and confidence intervals were computed and odds ratios (OR) for falls risk were calculated from exponentiated estimates, with 95% CIs reported for significant findings only. Underlying statistical assumptions were tested and verified. All statistical analyses were carried out using SAS Version 9.4.

## Results

421 Māori over 82 years and 516 non-Māori over 84 years were assessed at baseline, and 104 Māori (24.7%) and 198 non-Māori (38.3%) completed 5 years follow-up. Just over half (54%) of all LiLAC NZ survivors were enrolled in the study at Year 5 (Additional file 1: Table S1). Falls data were recorded at baseline for 408 Māori and 512 non-Māori which attenuated to 102 Māori and 197 non-Māori by Year 5. At baseline, 138 (33.8%) Māori and 205 (40%) non-Māori had fallen in the previous 12-months. Falls prevalence did not change markedly over the study, with comparable proportion for both cohorts other than for Years 3 and 5 when significantly more non-Māori fell. Māori were significantly younger than non-Māori by study design, because of the broader eligibility for age, and also presented with a higher body mass index (BMI) and higher scores for physical activity. MCR ctieria were met by 10 Māori and 7 non-Māori (Table 1).

**Table 1:** Baseline demographic & clinical outcomes, and falls status over 5 years

	Māori	non-Māori	*P*
	Mean (SD)	Mean (SD)	
Demographic and clinical outcomes^a^			
Age *M = 421; non-M = 516*	82.6 (2.7)	84.6 (.51)	**< 0.001**
BMI *M = 247; non-M = 375*	29.4 (5.4)	26.8 (4.0)	**< 0.001**
Gait speed (m/s) *M* = 243; non-*M* = 376	.66 (.29)	.82 (.26)	**< 0.001**
Gait speed (m/s) Men *M* = 96; non-*M* = 179	.70 (.25)	.87 (.27)	**< 0.001**
Gait speed (m/s) Women *M* = 147; non-*M* = 197	.64 (.30)	.78 (.25)	.099
Cognition: 3MS (maximum score 100) *M = 266; non-M = 393*	86.2 (15.6)	91.6 (10.4)	**< 0.001**
Depression: GDS_15 (range 0–15) *M = 255; non-M = 392*	2.5 (2.2)	2.1 (1.9)	**< 0.05**
Physical Activity: Total PASE score (maximum score 400) *M = 262; non-M = 403*	111.0 (85.0)	98.9 (66.0)	**< 0.05**
MCR (gait speed & GDS Item 10) *M = 238; non-M = 371* (%)	10 (4.2%)	7 (1.9%)	**< 0.001**
Falls status total n; falls n (%)			
Baseline	408; 138 (33.8%)	512; 205 (40%)	.055
Year 1	262; 90 (34.4%)	391; 160 (40.9%)	.101
Year 2	192; 54 (28.1%)	334; 121 (36.2%)	.068
Year 3	157; 53 (33.8%)	276; 121 (43.8%)	**< 0.05**
Year 4	116; 35 (30.2%)	228; 83 (36.4%)	.280
Year 5	102; 30 (29.4%)	197; 92 (46.7%)	**< 0.01**

^a^*Abbreviations*: *BMI* Body Mass Index, *3MS* Modified Mini Mental Status Examination, *GDS* Geriatric Depression Scale, *MCR* Motoric Cognitive Risk Syndrome, *PASE* Physical Activity Scale for the Elderly

^b^Student *t-*test for all comparisons other than MCR and falls status (χ2test)

Data in bold are significant

**Fig. 1:**
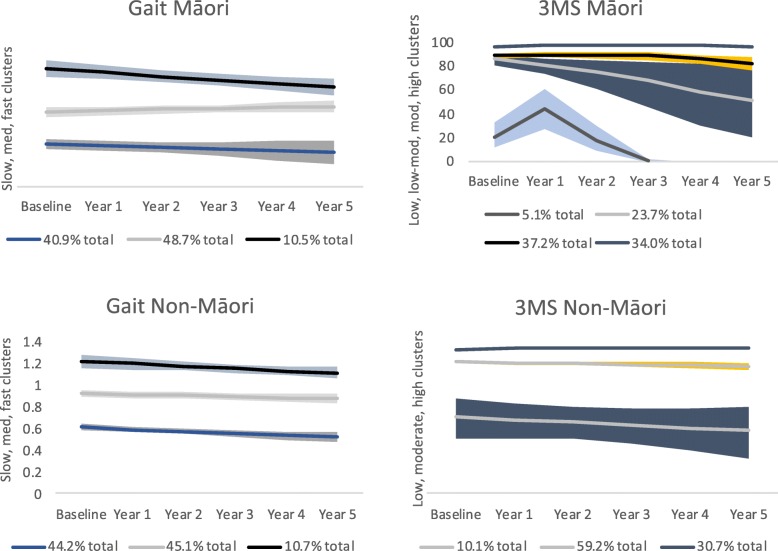
Clusters of Māori and non-Māori participants based on gait and cognition trajectories over 5 years. Frequency of participants in each cluster described as a percent of the total cohort

Gait speed was assessed for 243 Māori and 376 non-Māori, and cognition (3MS) completed by 266 Māori and 393 non-Māori. Non-Māori men walked significantly faster at baseline than Māori men and had higher scores for cognition (Table 1). Latent class analysis revealed 3 gait clusters for both cohorts (slow, medium, fast gait), 4 cognition clusters for Māori (low, low-moderate, moderate, and high) and 3 cognition clusters for non-Maori (low, moderate, high). Trajectories for gait and cognition differed for both cohorts (Fig. 1). For Māori, gait speed declined for the fast gait group who comprised 10.5% of the cohort. For all other Māori and for non-Māori gait speed was fairly stable. For cognition, Māori show a sharp decline between years 1 and 3 followed by cessation of data for the low cognition group who comprised 5% of the Māori cohort. Cognition also declined for the low-moderate group with 3MS scores almost 90/100 at Year 1, lowering to 60/100 and below by Year 5. The wide confidence intervals for this group indicate heterogeneity. Trajectories for the 2 highest scoring cognitive groups (over 2/3rds of the total Māori cohort) were stable over time. Compared with Maori, cognitive trajectories for non-Māori were overall more stable. CIs for the highest cognitive groups for Māori and non-Māori were very narrow for all years due to the left skew of the distribution, and are not visible in Fig. 1. For example, baseline Māori 95% CI was 96.4 [95.6, 97.1]; and baseline non-Māori CI was 97.9 [97.6, 98.2].

**Fig. 2:**
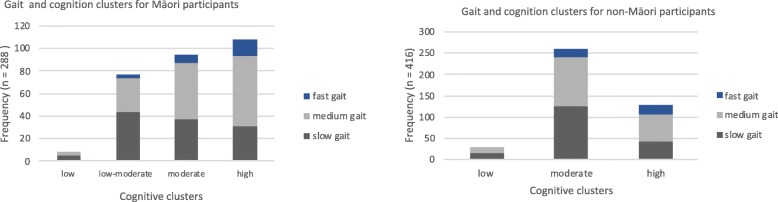
Frequency of participants in each gait and cognition cluster for Māori and non-Māori

Figure 2 shows the gait and cognition outcomes for each cluster. There were no fast walkers in the low cognitive group for either Māori or non-Māori, and only 8 Maori (2.8%) and 28 non-Māori (6.7%) participants met criteria for the ‘low cognition and slow gait speed’ group.

Table 2 shows the association between prospective falls and gender, age, gait and cognition scores. For the ‘gait and cogniton’ model where gait and cognition were entered as separate variables, gender was the only significant predictor of falls for Māori. Being male increased the odds of falling by 56% (OR 1.56; 95% CI 1.0–2.43 (*P* = 0.04). For non-Māori a slow gait significantly increased the odds of falling by 60% (OR 0.40; 95% CI 0.24–0.68) (*P* < 0.001). Lower 3MS scores also increased falls risk although this was not statistically significant (*P* = 0.07). For the ‘MCR model’ where MCR and cognition*gait interactions were included as predictors, there were no significant MCR findings for Māori. Non-Māori with MCR were more than twice as likely to fall than those without MCR (OR) 2.45; 95% CI 1.06–5.68 (*P* = 0.03). The interaction effects show that gait speed was a stronger predictor than cognition within this model (OR for gait 1.87. 95% CI 1.34–2.64 (*P* < 0.001) compared with cognition (OR 1.65; 95% CI 1.07–2.55); *P* = 0.02*.*

**Table 2:** Association of gait, cognition, and MCR with falls over 5 years

Predictors	Estimate	95% CI	OR^(a)^	*P*
*Māori gait & cognition model*^(a)^				
Gender (Male)	0.44	0.00 to 0.88	1.56	**0.04**
Age	0.04	−0.02 to 0.12	1.62	0.21
Cognition(3MS)	− 0.04	− 0.22 to 0.13	0.95	0.64
Gait speed (m/s)	0.18	−0.46 to 0.83	1.19	0.58
*Māori MCR model*^(a)^				
Gender (Male)	0.41	−0.85 to 0.20	0.66	0.06
Age	0.04	−0.02 to 0.12	1.05	0.20
MCR (subjective cognitive complaint and slow gait)	0.24	−0.87 to 1.35	1.27	0.67
Subjective cognitive complaint *normal gait	0.10	−0.38 to 0.60	1.11	0.66
No subjective cognitive complaint * slow gait	−0.33	−0.94 to0.27	0.71	0.27
*Non-Māori gait & cognition model*^(a)^				
Gender (Male)	0.12	−0.44 to 0.19	0.88	0.44
Age	0.05	−0.02 to 0.13	1.05	0.17
Cognition (3MS)	−0.11	−0.24 to 0.00	0.89	0.07
Gait speed (m/s)	−0.91	−1.44 to − 0.38	0.40	**< 0.001**
*Non-Māori MCR model*^(a)^				
Gender (Male)	0.15	−0.16 to 0.46	1.16	0.33
Age	0.05	−0.02 to 0.13	1.05	0.19
MCR (subjective cognitive complaint and slow gait)	0.89	0.05 to 1.73	2.45	**0.03**
Subjective cognitive compaint *normal gait	0.50	0.06 to 0.93	1.65	**0.02**
No subjective cognitive complaint * slow gait	0.63	0.29 to 0.97	1.87	**< 0.001**

^(a)^*Abbreviation*: *3MS* Modified Mini Mental Status Examination, *MCR* Motoric Cognitive Risk, *OR* calculated from exponentiated estimates

Data in bold are significant

Lastly, MCR prevalence for both cohorts was similar from baseline values (Table 1) until years 4 and 5 when no Māori presented with MCR, from 58 participants in Year 4 and 45 participants in Year 5 (data beyond baseline not reported in Table 1).

## Discussion

Main findings were that gait and cognition did not decline markedly over 5 years, a slower gait but not cognitive impairment significantly increased falls risk for non-Māori only, being male increaesd falls risk for Māori, and MCR prevalence was low especially for non-Māori. We rejected our hypothesis that gait and cognition would be independently associated with falls risk for both cohorts.

Of note are the high attrition rates for both cohorts, especially in the later years. Around 65% of the total sample was lost to follow-up, or death which although not surprising given the advanced age, will have undoubtedly biased results towards participants who were cognitively and motorically resilient, and who fell less. Kerse and colleagues report retention rates and reasons for drop out for the first 2 years of LiLACS, and note the higher drop-out for Māori for reasons other than death [12]. They also report significant differences in baseline characteristics for those retained in the study compared with those who died or were lost to follow-up.

Exceptions to the mostly stable scores for both cohorts over the 5 years were the 2 lowest cognitive scoring groups for Māori whose trajectory markedly declined, and who together comprised just over a quarter of the total Māori cohort. A further exception was the increase in the number of non-Māori fallers at Year 5. The modest decline in gait and cognition for those who remained in the study is an important finding and perhaps surprising given the multiple morbidities associated with advanced age, also evident for 93% of LiLAC NZ participants who reported at least 2 diagnosed health conditions at baseline [22].

Around a third of Māori and approximately 40% of non-Māori fell at any one time over the course of the study, which is broadly comparable with published data for adults over 65 years [23]. Non-Māori were slightly older which may have impacted on falls prevalence, especially by Year 5 where almost half non-Māori fell compared to under a third of Māori. Based on PASE scores at baseline, Māori were significantly more active than non-Māori, suggesting that lower falls prevalence was not related to lower amounts of activity. Falls prevalence for Māori did not increase over time, although the reasons for this are not clear.

Gait speed for non-Māori participants was comparable to norm-referenced data. Oh-Park [24] reports 0.95 m/s for men 80 to 84 years lowering to 0.88 m/s over 85 years, and for women 80 to 84 years 0.87 m/s lowering to 0.78 m/s over 85 years. Average gait speed for Māori was significantly slower, which may reflect anthropometric differences related to BMI which was significantly higher, or culturally different gait dynamics. Importantly, trajectories for gait speed for around 90% of Māori did not vary over the course of the study. Cognitive outcomes were considerably higher than published data for community dwelling older adults, for both cohorts. Bassuk and Murphy (2003) report an average 3MS score of 75.4 for people 75–84 years, lowering to 63.3 for those over 85 years. Only the 2 lowest Māori cognitive clusters had scores comparable to these, with all other groups scoring higher.

MCR prevalence at baseline was low particularly for non-Māori, although estimates are comparable to younger cohorts from Australia and UK [8]. Estimates for Māori were higher, although still lower than overall multi-country prevalence of 9.7% [9]*.* It is unwise to draw any conclusions from these results other than to note the low estimates probably reflect the high functioning nature of both cohorts, despite their advanced age. Kerse and colleagues documented the prevalence of dementia in LiLACS NZ as defined by 3MS scores (≤ 80 for Māori; ≤ 84 non-Māori), noting that the proportion of participants with no dementia stayed steady at approximately 87% [25]. Overall these findings point to a stable cohort with mostly preserved gait and cognition, both of which are critical to independent living and overall functioning.

Results from the multivariate modelling distingished between cohorts. For Māori, being male was the only significant predictor of falls when gait and cognition were entered separately into the model but not when MCR was included as an independent predictor. This may reflect a modifying effect of gender on gait speed. Māori men walked at a significantly slower pace than non-Māori men, but this was not the case for women. Earier reports suggest that being female confers a higher risk of falling [26], although data are not specific to adults of advanced years. For non-Māori, gait was a significant predictor of falls, and cognition trended towards significance. The findings were comparable when MCR criteria were included in the model. These differerences are interesting, although our interpretation is cautious in line with the small proportion with MCR. Results for non-Māori conform to a substantial body of research supporting the association between gait, cognition and falls in older adults [8, 27]. Results for Māori suggest the need for a broader repetoire of predictors to idenfity associations between falls and socio-cultural features such as whānau (family), living environment and support networks. These outcomes show that risk factors for falls are inconsistent across ethnic groups. Intervention strategies may also need rethinking.

The key strength of this study is the robust sampling which enabled reporting of novel data for a large cohort of Maori and non-Māori of advanced years with acceptable rates of attrition. Key limitations include sample bias as noted above, and the approach to collecting falls data which was retrospective via self-report and does not conform to best practice [15]*.* Also, gait speed was collected over a 3 m distance which is short, although congruent with population-based, longitudinal studies [28]. Lastly, because Māori were not involved in developing the outcomes for this study they are unlikely to reflect key aspects of Māoritanga (Māori culture, beliefs, traditions), and may therefore be culturally-biased towards non-Māori participants.

In conclusion this study contributes to our understanding of falls evolution and the role of gait and cognition to falls in community-dwelling adults of advanced age. For both Māori and non-Māori who live on into advanced years, fall frequency is relatively stable and there is minimal decline in gait and cognition For non-Māori, slow gait speed is a significant predictor of falls. These findings resonate widely, given current and projected demographic trends. Further work is needed to identify cultural appropriate strategies to improve falls risk for those living to advanced age.

## Supplementary information

**Additional file 1: Table S1.** LiLACS NZ survivors.

## Data Availability

The datasets used and/or analysed during the current study are available from the corresponding author on reasonable request. The process to apply for LiLACS NZ data access is available at https://www.fmhs.auckland.ac.nz/en/ faculty/lilacs.html, and we encourage applications to ensure the data gets maximal use for population health benefit. Each application is discussed by a leadership group of Māori and non-Māori academics.
