# Supplementary material for: Gait, cognition and falls over 5 years, and motoric cognitive risk in New Zealand octogenarians: Te Puāwaitanga o Nga Tapuwae Kia Ora Tonu, LiLACS NZ
Source: BMC Geriatr. 2020 Feb 5;20:43. doi: 10.1186/s12877-020-1420-8 (PMC7003444; doi:10.1186/s12877-020-1420-8)
Supplement: Supplementary file 1 — Additional file 1: Table S1. LiLACS NZ survivors. [file 12877_2020_1420_MOESM1_ESM.docx]

Table 1. LiLACS NZ survivors

| Cohort at inception | Māori Women  n = 245 | Māori Men  n = 176 | non-Māori Women  n = 279 | non-Māori Men  n = 237 | Total  n = 937 |
| --- | --- | --- | --- | --- | --- |
| Survivors at 5 years | 112 (46%) | 47 (27%) | 137 (49%) | 87 (37%) | 383 (41%) |
| Survivors at 5 years participating in LiLACS NZ | 49 / 112 (44%) | 20 / 47 (43%) | 81 / 137 (59%) | 55 / 87 (63%) | 205 / 383 (54%) |
